# Toll-like receptor 4 in acute viral infection: Too much of a good thing

**DOI:** 10.1371/journal.ppat.1007390

**Published:** 2018-12-20

**Authors:** Judith Olejnik, Adam J. Hume, Elke Mühlberger

**Affiliations:** 1 Department of Microbiology, Boston University School of Medicine, Boston, Massachusetts, United States of America; 2 National Emerging Infectious Diseases Laboratories, Boston University, Boston, Massachusetts, United States of America; University of Kentucky, UNITED STATES

Although a well-regulated inflammatory response is a vital defense mechanism against viral infection, too much inflammation can be detrimental. Excessive inflammatory responses, which are characterized by elevated levels of a broad array of pro-inflammatory cytokines and chemokines, have been observed in a wide variety of viral diseases associated with serious morbidity and mortality. Examples of this include acute lung injury caused by infections with respiratory syncytial virus (RSV), influenza A virus (IAV), or severe acute respiratory syndrome coronavirus (SARS-CoV). Excessive inflammatory responses induced by viral infections are not restricted to the lung but can be systemic, as reported for Ebola virus (EBOV) disease and severe dengue [1–5]. The reasons leading to an unbalanced inflammatory response in certain viral infections are not well understood and are most likely multifactorial. Here, we explore the role of toll-like receptor 4 (TLR4) in the induction of damaging inflammatory responses during acute viral infections.

## What is TLR4 and what are its ligands?

The innate immune system recognizes pathogen-associated molecular patterns (PAMPs) of viral or bacterial intruders via pattern recognition receptors (PRRs). This includes the family of TLRs that consists of related, transmembrane proteins that play a central role in the initiation of inflammatory responses, including the secretion of cytokines and chemokines.

TLR4, which is mainly expressed on cells of the immune system—including monocytes, macrophages and dendritic cells—has long been recognized as a PRR that senses lipopolysaccharide (LPS), a component of the outer membrane of gram-negative bacteria. Activation of TLR4 by LPS, its best studied ligand, is a multistep process. The initial step involves the LPS binding protein (LBP) which extracts LPS from bacterial membranes and LPS-containing vesicles to transfer it to the TLR4 coreceptor cluster of differentiation 14 (CD14). CD14 exists in two forms, soluble and membrane-bound. Both forms are able to interact with LPS-loaded LBP. CD14 breaks down LPS aggregates and transfers monomeric LPS into a hydrophobic pocket on myeloid differentiation factor 2 (MD-2) that is part of the MD-2/TLR4 complex. The high-affinity binding of LPS leads to dimerization and activation of the MD-2/TLR4 complex [6, 7]. Activation of TLR4 results in the recruitment of the intracellular adaptor protein, myeloid differentiation primary response 88 (MyD88), and/or toll/interleukin-1 receptor (TIR)-domain-containing adapter-inducing interferon-β (TRIF), ultimately resulting in the expression and secretion of pro-inflammatory mediators [6, 7].

TLR4 has also been shown to be a sensor for damage-associated molecular patterns (DAMPs). These include a wide variety of molecules released from injured or dying tissues as well as molecules actively released in response to cellular stress from intact cells [6, 8]. In addition to bacterial PAMPs and cellular DAMPs, TLR4 also recognizes PAMPs from other pathogens including fungi, parasites, and viruses [9]. How the TLR4 complex is activated by DAMPs and non-LPS PAMPs, which vary widely in their structure—some with no structural similarities to LPS [8, 10]—remains to be determined. Resolving the structure of these complexes is a critical part toward dissecting their mechanisms of activation.

## How do viruses activate TLR4?

There is a growing list of viruses that induce an inflammatory response during acute infection through TLR4 activation. Known TLR4-activating viral proteins include the RSV fusion protein (F), the EBOV glycoprotein, the vesicular stomatitis virus glycoprotein (VSV G), and the dengue virus (DENV) nonstructural protein 1 (NS1).

There are a number of commonalities between these viral TLR4 activators. For example, these proteins are all membrane-associated. VSV G, RSV F, and EBOV glycoprotein are classical viral glycoproteins that are exposed on the surface of viral particles and mediate fusion with host cell membranes through the hydrophobic fusion peptide. The fusion domain is only exposed after considerable conformational changes that occur at the plasma membrane (RSV F) or in the endosome (VSV G, EBOV glycoprotein). [11]. DENV NS1, although seemingly dissimilar to these surface glycoproteins, exists in multiple forms, including a secreted, membrane-bound form [12, 13]. The hydrophobic fusion peptide in RSV F has been suggested to bind into the deep hydrophobic pocket of MD-2, similarly to LPS, to mediate TLR4 activation [14]. TLR4 is stimulated by membrane-bound EBOV glycoprotein and a secreted, cleaved form (shed glycoprotein), both of which retain the hydrophobic fusion domain, but not by a different secreted version of EBOV glycoprotein—soluble glycoprotein—which lacks the fusion peptide [15, 16]. And although DENV NS1 lacks a fusion peptide, it contains exposed hydrophobic domains that mediate membrane interaction and could play a role in TLR4 activation [13]. TLR4 antagonists which suppress LPS-induced TLR4 signaling through competitive interaction with MD-2, such as LPS from the bacterium *Rhodobacter sphaeroides* (LPS-RS) and Eritoran, also suppress RSV F-, EBOV glycoprotein-, and DENV NS1-mediated TLR4 activation [12, 14, 17–20], suggesting similar mechanisms of action. However, it remains to be determined how these large glycoproteins interact with the TLR4 receptor complex and in what way the hydrophobic regions would be accessible for interaction with MD-2 to potentially activate TLR4 signaling.

VSV G, RSV F, EBOV glycoprotein, and DENV NS1 are all glycosylated. Although it is possible that the glycosylation of these proteins is merely a coincidence because many membrane-bound proteins are glycosylated, this raises the question of whether glycosylation is a general feature that is required for viral TLR4 activation. Indeed, LPS glycan structures play an essential role in regulating different steps in TLR4 activation. This includes increasing the stability of the LPS-MD-2/TLR4 complex via direct interaction of LPS core saccharides with TLR4 [21–23]. As for the viral TLR4 activators, glycosylation of EBOV glycoprotein is required for TLR4 activation [16, 24], but it is not known whether this is also the case for the other viral glycoproteins.

Unlike the viruses mentioned above, IAV does not activate TLR4 by a specific viral protein but rather induces TLR4 activation by host DAMPs, including high-mobility group box 1 protein (HMGB1) and oxidized phospholipids, which accumulate in response to infection [25, 26]. HMGB1, which is also a glycoprotein, activates TLR4 through MD-2 binding [8, 27]. Whereas host DAMPs might play a central role in acute lung injury and are detected in the lungs of patients with severe IAV or SARS-CoV infections [26], the role of DAMP-mediated TLR4 activation in other viral infections remains largely unexplored.

## Is TLR4 activation during acute viral infections beneficial or harmful?

**Fig 1:**
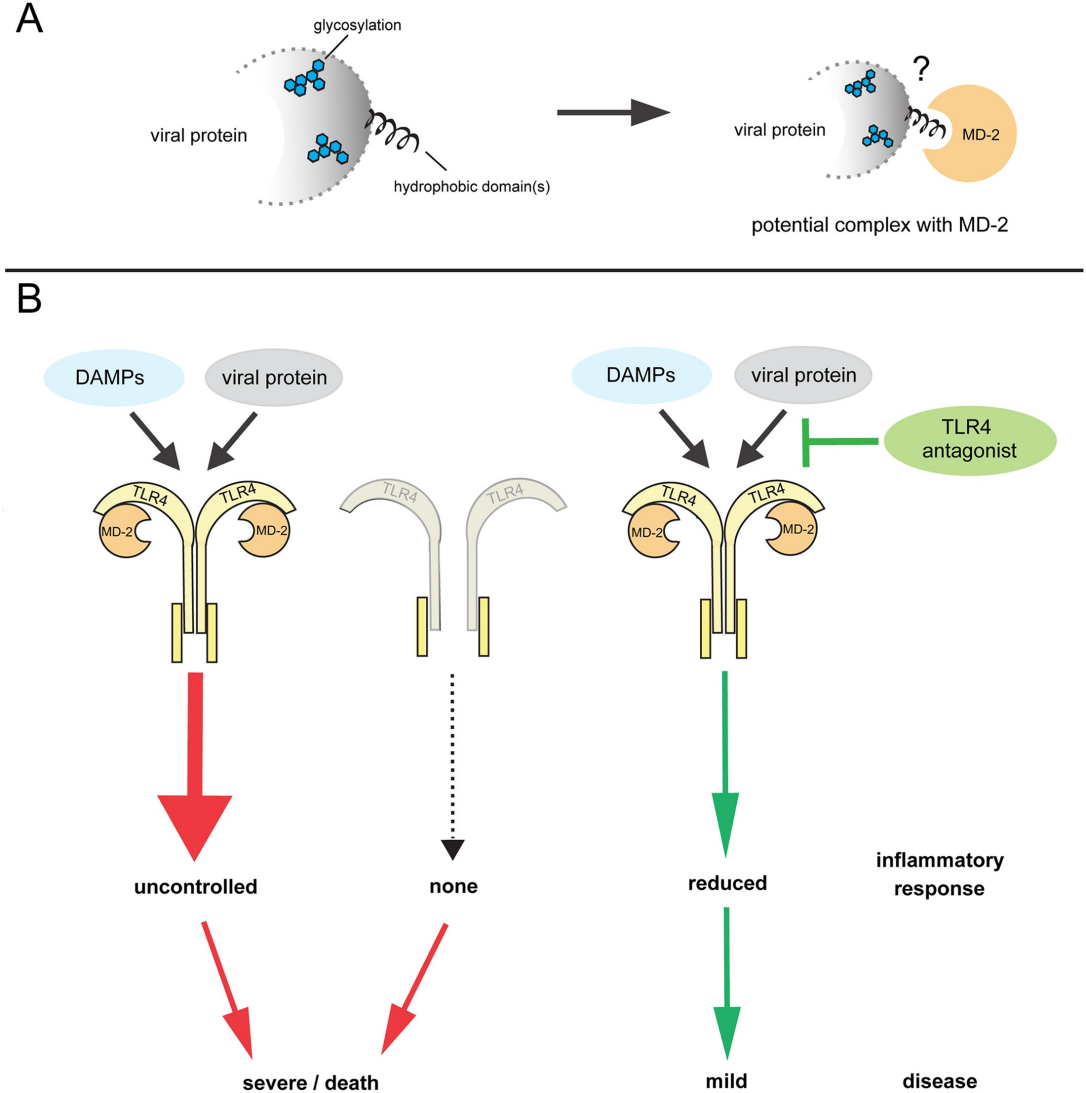
TLR4 in viral pathogenesis. (A) Viral proteins known to activate TLR4 are membrane-bound or membrane-associated, contain hydrophobic domains, and are glycosylated. Although it remains to be determined how these viral proteins interact with the MD-2/TLR4 complex, data from known TLR4 activators suggest that the hydrophobic domains of these viral proteins might bind in the hydrophobic pocket of MD-2. Glycans on the viral protein could be involved in stabilizing the MD-2/TLR4 complex to enhance TLR4 signaling. Compared to the well-described interaction of TLR4 complexes with relatively small bacterial LPSs, the mechanism by which these complexes recognize large viral glycoproteins to trigger downstream signaling remains largely unexplored. (B) Viral proteins and host DAMPs, which accumulate in response to cellular stress during viral infection, have been linked to TLR4 activation during virus infection. Both uncontrolled activation of TLR4 and TLR4 knockout are associated with severe disease, whereas reducing the TLR4-mediated inflammatory response using TLR4 inhibitors mitigates disease symptoms, offering potential treatment options for various severe viral infections. DAMP, damage-associated molecular pattern; LPS, lipopolysaccharide; MD-2, myeloid differentiation factor 2; TLR4, toll-like receptor 4.

Treatment with TLR4 antagonists has consistently resulted in reduced cytokine and chemokine production and mitigated disease symptoms in small animal models of IAV, EBOV, and DENV infections [12, 19, 25, 28, 29], clearly identifying a role for TLR4 activation in the pathogenesis of these viral diseases. Lethal infection of mice with EBOV and IAV was prevented by treatment with TLR4 antagonists, highlighting the therapeutic potential of these compounds [19, 25, 28, 29]. The picture becomes more complicated, however, when TLR4 knockout mice were used. Mice lacking TLR4 had either similar survival rates or even more severe disease than wild-type mice infected with DENV, EBOV, SARS-CoV, or RSV [19, 20, 30, 31]. Infection of TLR4 knockout mice with IAV resulted in a variety of outcomes [32], possibly due to variations in the genetic background of the mouse strains and the use of different experimental systems, including the analyzed time points. Altogether, data from TLR4 knockout mice suggest that protective immune responses against these viruses might require some degree of TLR4 activation (Fig 1).

The role of TLR4 activation in EBOV disease is particularly noteworthy. In contrast to the highly pathogenic EBOV, Reston virus (RESTV), a member of the *Ebolavirus* genus that is believed to be nonpathogenic for humans, lacks the ability to significantly stimulate TLR4, providing further evidence that TLR4 activation contributes to EBOV pathogenicity [17]. When nonhuman primates were infected with EBOV stocks of equal titers, the stock with a higher particle-to-plaque−forming unit (PFU) ratio was associated with increased disease severity [33]. A possible explanation for this observation could be that glycoprotein exposed on the surface of noninfectious EBOV particles activates TLR4, thereby enhancing the damaging inflammatory response. Finally, shed glycoprotein was detected at high levels in EBOV-infected guinea pigs, particularly shortly before death [34]. Shed glycoprotein is sufficient to stimulate cytokine responses in the absence of infection [16], suggesting that noninfected TLR4-expressing cells stimulated by shed glycoprotein might contribute to the complex inflammatory response in EBOV disease [15].

## TLR4 activation: What are the benefits for the viruses?

Given the high mutation rate of RNA viruses that enables them to escape from challenging host responses, the question arises whether the activation of TLR4 is beneficial for a productive viral infection. One potential benefit to activating TLR4 during viral infection might be to induce specific host factors that promote viral replication or repress those with antiviral activity. There is evidence for this as TLR4 activation during EBOV infection increases the expression of suppressor of cytokine signaling 3 (SOCS3), which has been shown to enhance viral particle release [17, 35]. Phosphatidylinositol-4,5-bisphosphate 3-kinase (PI3K), a cell survival factor that prevents premature apoptotic cell death, is activated during infection with IAV, RSV, DENV, and SARS-CoV [36], and PI3K activation can be mediated by TLR4 [37]. TLR4-induced innate immunity could further skew adaptive immune responses in a manner that favors viral replication. This is speculative, however, and additional research is needed to understand the effects of TLR4 activation on viral propagation.

## Conclusion

In conclusion, activation of TLR4 seems to play a nuanced role during viral infection. Although over-stimulation of TLR4 can lead to an excessive inflammatory response that is damaging to the host, a certain amount of TLR4 activation may be beneficial to the host by helping to establish a protective immune response. Studies with well-known TLR4 inhibitors convincingly show that dampening the excessive inflammatory response mitigates disease and promotes survival, highlighting the therapeutic potential of TLR4 inhibitors in viral infections. More work is needed to dissect the mechanisms by which large viral glycoproteins activate TLR4. Several questions of particular interest remain: are glycan structures or hydrophobic domains on viral TLR4 activators required for activation? Could lipid modifications contribute to MD-2/TLR4 activation? What are the factors leading to uncontrolled cytokine release versus a balanced TLR4 activation conferring protective immune responses? Considering the devastating inflammation induced by these viruses, determining how and why these viruses activate TLR4 may be critical not only for understanding why these pathogens cause severe disease but also for the development of effective antiviral therapies.
